# Mouse Models of the Skin: Models to Define Mechanisms of Skin Carcinogenesis

**DOI:** 10.1155/2013/971495

**Published:** 2013-06-02

**Authors:** Deric L. Wheeler, Ajit K. Verma, Mitchell F. Denning

**Affiliations:** ^1^Department of Human Oncology, School of Medicine and Public Health, University of Wisconsin, Madison, WI 53705, USA; ^2^Department of Pathology, Stritch School of Medicine, Loyola University Chicago, Chicago, IL 60153, USA

The multistep model of mouse skin carcinogenesis has facilitated identification of irreversible genetic events of initiation and progression, and epigenetic events of tumor promotion. Mouse skin tumor initiation can be accomplished by a single exposure to a sufficiently small dose of a carcinogen, and this step is rapid and irreversible. However, promotion of skin tumor formation requires a repeated and prolonged exposure to a promoter, and that tumor promotion is reversible. Investigations focused on the mechanisms of mouse carcinogenesis have resulted in the identifications of potential molecular targets of cancer induction and progression useful in planning strategies for human cancer prevention trials. This special issue contains eight papers that focus on mouse models used to study individual proteins expressed in the mouse skin and the role they play in differentiation, tissue homeostasis, skin carcinogenesis, and chemoprevention of skin cancer. 

In the paper entitled “*Ap1 transcription factors in epidermal differentiation and skin cancer*,” R. Eckert et al. highlight the role of AP1, a transcription factor composed of c-jun and c-fos, that serves as a central node in epidermal keratinocyte survival and differentiation. The authors discuss how AP1 deregulation leads to key steps in driving the development of cancer and how these functions in cancer may be different in epidermal development. Finally, they summarize the various mouse models that have helped elucidate the role of this very interesting molecule. 

In the paper entitled “*The role of TGF*β* signaling in squamous cell cancer: lessons from mouse models*,” A. Glick summarizes the current literature on the role of TGF*β*1 in normal tissues and in carcinogenesis. TGF*β*1 is a member of a large growth factor family including activins/inhibins and bone morphogenic proteins (BMPs) that have potent growth regulatory and immunomodulatory functions in normal skin homeostasis, regulation of epidermal stem cells, extracellular matrix production, angiogenesis, and inflammation. The author presents a thorough comparison between the role of TGF*β*1 in signaling in human HNSCC and cutaneous SCC and the various mouse models that have been developed to elucidate the role this molecule plays in oncogenesis.

In the paper entitled “*Multiple roles for VEGF in non-melanoma skin cancer: angiogenesis and beyond*,” K. Johnson and T. Wilgus overview how vascular endothelial growth factor (VEGF), a potent proangiogenic factor in mouse and human skin tumors, plays a role in the development of non-melanoma skin cancers. The authors have detailed the use of both transgenic and knockout mice that have provided key clues, primarily alteration of proliferation, survival, and stemness, that have helped elucidate the function of VEGF in carcinogenesis. 

In the paper entitled “*Protein kinase C*ε*, which is linked to ultraviolet radiation-induced development of squamous cell carcinomas, stimulates rapid turnover of adult hair follicle stem cells*,” A. Singh et al. report that protein kinase C epsilon (PKC*ε*), a member of the protein kinase C superfamily, plays a critical step in the development of cutaneous SCC induced by repeated exposures to ultraviolet radiation (UV). The authors focus their investigation on how PKC*ε*, using transgenic mice, may modulate the hair follicle stem cell (HSC). The authors report that overexpression of PKC*ε* in the skin, driven by the K14 promoter, leads to a 7-fold increase in the proliferation of the HSC, indicating a rapid turnover of these cells. 

In the paper entitled “*Patched knockout mouse models of basal cell carcinoma*,” the authors discuss the link Patched (PTCH), the receptor for the hedgehog ligand, in the development of Basal cell carcinoma (BCC), the most common form of human skin cancer. In this comprehensive review, the authors compare conventional and conditional PTCH knockout mouse models to investigate BCC as well as for potential use in preclinical research.

In the paper entitled “*Delineating molecular mechanisms of squamous tissue homeostasis and neoplasia: focus on p63*,” K. King et al. focus on summarizing mouse models that have highlighted the importance of p63, a transcription factor that plays an essential role in the development and maintenance of normal stratified squamous epithelium. The authors present that p63 has multiple splice variants and p63 plays a critical role in normal skin biology and neoplastic development.

In the paper entitled “*Role of Stat3 in skin carcinogenesis: insights gained from relevant mouse models*,” E. Macias et al. review the role of signal transducer and activator of transcription 3 (Stat3) in skin biology. The authors detail the various transgenic, knockout, and conditional knockout mice that have led to the understanding of STAT3 in normal skin homeostasis, migration, wound healing, and hair follicle growth and maintenance as well as skin carcinogenesis. 

In the paper entitled “*Topical curcumin-based cream is equivalent to dietary curcumin in a skin cancer model,” *the authors present the first study that compares the use of topical curcumin versus the use of oral curcumin as a chemopreventive strategy for the development of SCC of the skin.

This collection of papers provides an overview of mouse models investigating several intensely studied molecules involved in skin carcinogenesis. We hope the molecular mechanisms revealed in this special issue will enlighten readers and provide them with motivation to continue their research endeavors.

*Deric L. Wheeler* *Deric L. Wheeler* *Ajit K. Verma* *Ajit K. Verma* *Mitchell F. Denning* *Mitchell F. Denning*

